# Low Cross-Sex Genetic Correlation in Carotenoid-Based Plumage Traits in the Blue Tit Nestlings (*Cyanistes caeruleus*)

**DOI:** 10.1371/journal.pone.0069786

**Published:** 2013-07-25

**Authors:** Szymon M. Drobniak, Dariusz Wiejaczka, Aneta Arct, Anna Dubiec, Lars Gustafsson, Mariusz Cichoń

**Affiliations:** 1 Institute of Environmental Sciences, Jagiellonian University, Kraków, Poland; 2 Museum and Institute of Zoology, Polish Academy of Sciences, Warszawa, Poland; 3 Department of Animal Ecology, Evolutionary Biology Centre (EBC), Uppsala University, Uppsala, Sweden; Arizona State University, United States of America

## Abstract

In some bird species, both adult and juvenile individuals are often brightly coloured. It has been commonly assumed that identical plumage colouration present in both sexes results from strong intersexual genetic correlations in colour-related traits. Here, we aimed at testing this hypothesis in juvenile individuals and looked at genetic parameters describing carotenoid-based colouration of blue tit nestlings in a wild population. To separate genetic and environmental sources of phenotypic variation we performed a cross-fostering experiment. Our analyses confirmed the existence of sexual dichromatism in blue tit nestlings and revealed a significant, although low, genetic component of carotenoid-based colouration. However, genetic effects are expressed differently across sexes as indicated by low cross-sex genetic correlations (*r_mf_*). Thus our results do not support the prediction of generally high *r_mf_* and suggest that intersexual constraints on the evolution of colouration traits may be weaker than expected. We hypothesise that observed patterns of genetic correlations result from sex-specific selective pressures acting on nestling plumage colouration.

## Introduction

Genetic correlations (*r_g_*) describe the strength of a relationship between two traits at the genetic level, and usually predict their concerted evolution [1]. Genetic correlations are usually considered for pairs of different traits, they can however also describe genetic relationships within a single trait expressed in different environments or sexes. It is obvious that males and females should share 100% genes on their autosomes. Thus, assuming lack of sex-chromosome linkage, one should expect strong intersexual correlations (*r_mf_*) between genes expressed in males and females [1], [2], [3]. Such correlation constitutes a null genetic model for the studies of intersexual genetic correlations [4].

Intersexual genetic correlations – if present – are likely to constrain evolutionary potential of one or both sexes [5], [6]. However, strong intersexual *r_mf_* does not always lead to sexual conflict constraining evolutionary processes: strong positive correlation between the two sexes will constrain evolutionary change if the directions of selection in the separate sexes are opposite (i.e. bivariate selection vector is (close to being) perpendicular to the axis of maximum genetic variance; [7]). Such contrasting selection is usually observed in the form of sex-specific selection [3], [6], [8]. Commonness of sexual dimorphism (SD) in many animal taxa seems contradictory to the expectation of generally strong and positive cross-sex *r_mf_* [2], [6]. An array of mechanisms has been suggested to explain this apparent paradox [9]. The majority of them assume modification of traits’ genetic architectures resulting in lowered *r_mf_* and relaxed genetic constraints [6], [10], [11], [12]. Indeed, [3] provides empirical support for the importance of low *r_mf_* for the evolution of sexual dimorphism by showing that the magnitude of dimorphism and *r_mf_* are negatively correlated.

Virtually all published studies on sexual dimorphism focus on adult individuals and clearly favour sexual selection as the preferred explanation of SD. However, accumulating evidence suggests that sexually dimorphic traits are also observed in juvenile, non-reproducing individuals, on which sexual selection is unlikely to operate. Such phenomenon is best known in several bird species [13], [14], [15], [16]; see also [9]. One type of dimorphism – i.e. dichromatism – is particularly interesting in this case: brightly coloured feathers expressed by nestlings are entirely or almost entirely moulted before the first breeding attempt, which makes sexual selection an unlikely explanation of the observed colour dimorphism [17], [20]. Comparison of genetic architecture of juvenile and adult traits could shed some light on the evolutionary processes involved in the evolution of juvenile SD –however such studies are lacking. It is particularly interesting whether dimorphic traits expressed in juveniles are indeed associated with low *r_mf_* (as often observed in adult individuals [3]). In fact such observation would indicate that – although expressed in the absence of sexual selection – dimorphic juvenile traits have similar genetic architecture as adult traits. [21] recently addressed the problem of the plasticity of genetic correlations between sexes during ontogeny. Although their review demonstrated that intersexual genetic correlations tend to decrease over the lifetime of an individual, their discussion did not address the issue of dimorphic juvenile traits. Thus, it is still unclear whether juvenile traits expressing similar dimorphism as adult traits exhibit similar, low levels of cross-sex genetic correlations.

Our aim was to explore genetic patterns in sexually dimorphic traits in blue tit (*Cyanistes caeruleus*) nestlings. In this species, both adults and juveniles express yellow colouration of breast feathers [17], which is based on the deposition of various carotenoids, mainly lutein and zeaxantin [18]. The juvenile breast plumage colouration shows distinct sexual dimorphism [15]. Importantly, all yellow feathers are moulted entirely before maturation, within a few months after fledgling [17]. Thus, juvenile plumage in this species does not seem to be exposed to sexual selection. However, based on the clear sexual dimorphism observed in juvenile plumage, we predict to find a low intersexual genetic correlation in nestling carotenoid-based colouration.

## Materials and Methods

### General Methods

The study was conducted in 2007 and 2008 in the population of blue tits breeding in nest-boxes on the Swedish island – Gotland (57°03′N, 18°17′E) (see [22] for a more detailed description of the study area).

From the end of April we regularly inspected nest-boxes. For each brood, number of eggs, date of laying and date of hatching (day 0) were recorded. Nestlings were uniquely marked by nail clipping on day 2 followed by ringing on day 11. Two days post-hatching nestlings were blood-sampled for molecular sex identification (see [23] for details) and half of the brood was cross-fostered within a dyad of nests containing equal number of hatchlings (±1) of the same age. These nests were also subjects to a brood-size manipulation experiment – some (randomly selected; one nest in every dyad) nests were enlarged by adding 3 additional nestlings (not related to the cross-fostered families, not included in further analyses) and other were left unmanipulated. Donor broods from which chicks were moved to enlarge experimental nests were excluded from all analyses. The effect of this manipulation was considered in all analyses to account for possible influence of brood enlargement on colour traits. In total, we analysed 50 nests forming 25 complete dyads, containing together 594 nestlings.

Breast feathers’ colouration was measured as described in [24]. Briefly, up to 10 feathers were taken from both sides of a nestling’s breast (14^th^ day post-hatching) and placed between two glass slides together with colour standards helping to calibrate subsequent colour sampling. The arrangement of feathers resembled that on the living bird in such a way that they formed two superimposed layers. Samples were photographed (Canon 400D with Canon MP-E 1–5× Macro Lens; distance from the lens to the sample: 25 cm) in a black-box using an unidirectional halogen light source. Photographs were analysed blindly with respect to the origin of the sample using Colour Analysis Tool software (http://wwwfxc.btinternet.co.uk). We averaged RGB colour values from ten 2×2 mm squares per individual. Measurements were highly repeatable (Table S1 in File S1). RGB values were converted into hue (perceived colour tone), brightness (total amount of reflected light) and saturation (intensity of colour) using the formulas from [25].

Described method of colour quantification is likely to yield measures not corresponding directly to the way birds perceive colours [18], [26]. Birds have four types of photoreceptors, including one for ultra-violet light [27], which is impossible to be measured using photographic methods. However, a number of studies have shown that such measures of colour provide important, biologically relevant information and should be considered as valid if they are standardised, repeatable and blind with respect to sample identity [14], [19], [28]. As pointed out by [29] “for heuristic purposes, it may be useful to express colour patterns in subjective terms that humans can readily understand”. As carotenoids absorb light mainly in the part of the spectrum visible to the human eye, we believe that our measurements accurately reflect this component of colour signal expressed in the plumage of blue tits. It is of course possible that the UV component constitutes an important part of carotenoid-based signal. Thus, interpretation of all results presented here relies on the fact that we analysed only information directly related to the concentration of respective carotenoids. Such an approach is also supported by a recent study suggesting, that UV-part of a carotenoid-based ornament contains no additional information about the concentration of carotenoids deposited in feathers [37].

### Ethics Statement

This study was performed with accordance to the ethical regulations of the Swedish Research Council (ethical permit nr S-53-11). We made all efforts to minimize the time required to handle the nestlings and thus to decrease the stress associated with all procedures.

### Statistical Analyses

Colour data were analysed with using linear mixed models implemented using the Markov Chain Monte Carlo method in R 2.9.2 [30] (MCMCglmm package, [31]). We used two sets of models: (i) univariate models and (ii) bivariate formulations of the previous, with male and female traits included as separate dependent variables. Three models were fitted in each of these two sets, with respect to all three colour variables (hue, saturation, brightness) as dependent variables.

The models fitted experimental treatment (brood-size manipulation) and year as fixed effects. Additionally, sex was introduced in the univariate models. All models contained random effects of nest of origin, nest of rearing and dyad. Inclusion of each of these effects was decided upon the deviance information criterion (DIC) (Table S2 in File S1). DIC is commonly used in MCMC-based analyses as a simple measure of goodness of fit, similarly to AIC in REML framework. Here we preferred the use of DIC instead of variance components confidence intervals (CI) as in MCMCglmm variance estimates are forced to be positive, which makes CIs inappropriate for testing significance of random effects. Unlike variances, hypotheses related to covariances (and correlations) were tested using CIs as covariances (and correlations) are not restricted to positive values [31]. All reported CIs are Bayesian highest posterior density intervals (95% credibility intervals).

Using such kind of full-sib analyses assumes that there is no relatedness between the analysed families. In such a case, nest-of-origin approximates half of additive genetic variance plus quarter of dominance variance and maternal effects (ME) if present [1]. Variance component related to nest of origin was used to calculate broad-sense heritability (*H^2^*). Since we defined sex-specific variances for nest-of-origin effect we were able to estimate also related covariances. Dividing genetic covariance by geometrical mean of the respective variances yielded genetic correlations [1].

All analyses were checked for possible problems resulting from autocorrelations in MCMC time series by visual inspection of the time-series plots and by the calculation of autocorrelations between successive samples. No autocorrelation-related issues were detected in any of the models. All models were fitted with relatively uninformative, inverse-Wishart distributed priors (variance *V* = 1, covariance *COV* = 0, *ν* = 1.002). However, all models were robust to prior modification – changing priors by doubling variances had no effect on qualitative conclusions drawn from our analyses.

### Methodological Issues

Interpretation of our results relies on one important assumption. Our estimates of variance components assume all nestlings in a single nest-of-origin to be full-sibs, which might not be true. Blue tits in our population exhibit extra-pair paternity (EPP) of about 8% (proportion of nestlings from extra-pair matings in the whole population, unpublished data). Thus, our estimates of heritability and genetic correlations might be downwardly biased. It is hard to quantify the strength of this bias as, to our knowledge, the influence of misassigned paternities on the estimates of heritability and genetic correlations has rarely been rigorously assessed [32]. Estimates based on simulations show that the frequency of EPP observed in our population should not considerably bias estimates of heritability [32]. Thus, it is very unlikely that estimates presented in this paper are seriously affected by neglecting variation related to EPP.

## Results

We found evidence for sexual dimorphism in all three colour-related nestling traits: males were on average brighter, more saturated (although here intersexual difference was marginally non-significant) and yellower (univariate analysis, Table S2 in File S1). The effects of experimental treatment (enlarged vs. control broods) appeared significant in the case of hue and saturation, whereas the effect of sex was significant in case of brightness and hue (univariate analyses, Table S2 in File S1). Variance components associated with nest-of-origin, nest-of-rearing and dyad appeared significant in all models (Table S3 in File S1). We present both DIC values for all models considered (Table S3 in File S1) and 95% CIs (Table 1). Thus, there is a significant genetic component of carotenoid-based plumage of blue tit nestlings (Table 1). Heritability of carotenoid-based colouration is significant, although relatively low (H^2^ for colour related traits reaches 8% (95% CI: lower 4%, upper 18%)), and does not differ significantly between sexes (see respective CIs in Table 1). The bivariate analyses revealed weak genetic link between male and female ornamentation in blue tits as indicated by low intersexual genetic correlations (*r_mf_*) in all three colour traits, with *r_mf_* for brightness being even weakly negative (Table 1). These estimates of genetic correlations appeared not significantly different from zero and significantly different from unity, as indicated by respective confidence intervals.

**Table 1:** Variance/covariance estimates and their confidence intervals (in brackets).

Model type	Random effect	Brightness	Saturation	Hue
Univariate model	O	0.46 (0.21;1,18)	0.57 (0.21;1.20)	0.08 (0.04;0.21)
		H^2^ = 0.08 (0.04;0.18)	H^2^ = 0.08 (0.03;0.18)	H^2^ = 0.05 (0.02;0.13)
	R	0.65 (0.23;1.40)	0.58 (0.26;1.58)	0.16 (0.06;0.33)
	D	0.48 (0.20;1.28)	0.71 (0.22;1.57)	0.10 (0.04;1.24)
	Res	10.65 (9.51;12.14)	10.10 (9.15;11.81)	2.79 (2.44;3.11)
Bivariate model	O M	1.11 (0.41;2.34)	0.79 (0.41;2.29)	0.25 (0.12;0.55)
		H^2^ = 0.14 (0.05;0.29)	H^2^ = 0.13 (0.06;0.30)	H^2^ = 0.11 (0.05;0.20)
	O F	1.07 (0.39;2.38)	1.07 (0.46;2.40)	0.16 (0.07;0.32)
		H^2^ = 0.17 (0.07;0.37)	H^2^ = 0.17 (0.08;0.36)	H^2^ = 0.18 (0.08;0.33)
	O MF	0.02 (−0.66;0.8)	0.12 (−0.55;0.98)	0.01 (−0.11;0.16)
		r_mf_ = −0.13 (−0.50;0.51)	r_mf_ = 0.19 (−0.36;0.64)	r_mf_ = 0.16 (−0.42;0.67)
	R M	1.17 (0.44;2.61)	1 (0.49;2.58)	0.42 (0.15;0.89)
	R F	0.93 (0.39;2.46)	1.3 (0.58;2.71)	0.2 (0.07;0.39)
	D M	0.68 (0.22;2.28)	0.72 (0.32;2.3)	0.21 (0.07;0.38)
	D F	0.57 (0.22;2.07)	0.73 (0.27;2.04)	0.1 (0.07;0.46)
	Res M	11.06 (9.43;13.23)	10.26 (8.92;12.74)	4.2 (3.58;5.05)
	Res F	8.45 (7.3;10.11)	8.91 (7.38;10.36)	1.3 (1.06;1.48)

For nest-of-origin variance/covariance broad-sense heritabilities (*H^2^)* and cross-sex genetic correlations (*r_mf_*) are presented together with respective CIs. Random effects: O – nest-of-origin; R – nest-of-rearing; D – dyad; Res – residual variance; M/F – male/female specific effects; MF – indicates covariance between males and females.

## Discussion

Our analysis revealed that the colour of breast feathers’ is heritable in the blue tit nestlings, although the contribution of the genetic component in the total observed variance is relatively low. Our estimates of heritabilities are similar to published estimates for blue and great tit nestlings [18], [24], [33] and add to a growing body of evidence that carotenoid-based plumage characteristics are weakly heritable. Estimated heritabilities are moderate to low, indicating that environmental effects play an important role in shaping total phenotypic variance in carotenoid-dependent colouration of blue tit nestlings [26], [34].

Nestling ornamentation is usually assumed to result from selection other than sexual selection [15], [20]. Assuming that non-sexual selection is less likely to produce sex-specific selective pressures, one would expect such juvenile traits to lack sex-specificity. In our study genetic parameters lacked any sex-specificity, i.e. we observed no differences in heritabilities between sexes. However, we found that male and female blue tit nestlings are not correlated genetically with respect to their carotenoid-based colouration, i.e. variation in their ornamentation is largely influenced by a genotype-by-sex interaction. Presence of such low *r_mf_* suggests recent sexual conflict usually generated by sex-specific disrupting selection [6], [8]. Since sexual selection acting on juvenile birds is not likely, there must be some other explanations of the observed lack of genetic intersexual correlations in nestling colour traits.

Firstly, juvenile plumage may be ontogenetically correlated with that of adult birds. In such a case juvenile plumage would be a mere dysfunctional side-product of selection acting on adult individuals – and all genetic patterns observed in offspring plumage would in fact reflect underlying genetics of adult traits. Usually, strong genetic correlations between analogous traits expressed throughout the ontogeny are expected [9]. However, concluding that low *r_mf_* in blue tit nestlings results from (sexual) selection acting on adults would require demonstrating that juvenile and adult plumage traits are strongly correlated genetically. Compelling evidence for such correlations in blue tits does not exist. Only one study estimated phenotypic juvenile-adult correlation (i.e. within the same individuals) in carotenoid-based plumage traits in a closely related species – the great tit - [19] and failed to show such correlation. Our unpublished data also do not support such correlation. However more robust and unequivocal estimates of the genetic juvenile-adult correlations in plumage traits are required to reject this explanation.

Secondly, carotenoid-based plumage colouration in juvenile blue tit might be adaptive at this stage of life independently of its function in the adulthood. It is unlikely that plumage colouration plays any role in the competition inside a nest or in parental favouritism as tits breed in cavities with limited light availability [20]. Plumage colouration may however play an important role in the post fledgling period. Several hypotheses have been proposed here. Some suggest that juvenile colouration may serve as a signal in establishing social hierarchy in the flock [35], [36]. Other suggest that juveniles might use carotenoid-coloured plumage as signals of their individual quality to their parents during the post-fledgling parental care period [20]. The extent to which these mechanisms may be sex-specific – and as such give rise to lower *r_mf_* – remains to be studied.

To conclude, our results provide first evidence that genetic constraints on the independent evolution of male and female ornamentation observed among nestlings may be weak. Lack of strong intersexual genetic correlation is contrary to a common assumption of shared genetic background of the sexes. However, the potential sex-specific selection forces responsible for diminishing the expected genetic correlation between sex remains to be identified. Further studies, focusing on sex-specific function of carotenoid-based plumage colouration in juveniles, should explain evolutionary mechanisms that produce low cross-sex genetic correlations among nestlings, and will certainly open new and exciting research perspectives.

## Supporting Information

File S1 **Tables S1–3 providing repeatabilites of analysed traits, fixed-effects estimates and DIC-based model selection.**
(DOC)
