# Supplementary material for: Low Cross-Sex Genetic Correlation in Carotenoid-Based Plumage Traits in the Blue Tit Nestlings (Cyanistes caeruleus)
Source: PLoS One. 2013 Jul 25;8(7):e69786. doi: 10.1371/journal.pone.0069786 (PMC3723658; doi:10.1371/journal.pone.0069786)
Supplement: File S1 — Tables S1–3 providing repeatabilites of analysed traits, fixed-effects estimates and DIC-based model selection. (DOC) [file pone.0069786.s001.doc]

Table S1. Repeatability of colour measurements both within-sample and within-individual.

| Colour component | Within-sample repeatability | | | Within-individual repeatability | | |
| --- | --- | --- | --- | --- | --- | --- |
|  | r | F_df1,df2_ | P | r | F_df1,df2_ | P |
| Red | 0.83 | 57.61_685,1373_ | <0.0001 | 0.81 | 52.33_200,402_ | <0.0001 |
| Green | 0.83 | 54.38_685,1373_ | <0.0001 | 0.80 | 47.28_200,402_ | <0.0001 |
| Blue | 0.81 | 49.72_685,1373_ | <0.0001 | 0.80 | 46.42_200,402_ | <0.0001 |

Table S2. Results of univariate analyses for fixed effects, presented as respective estimates and 95% confidence intervals. Experiment stands for brood-size manipulation. The interpretation of estimates is conventional, i.e. intercept represents females in the control group and the remaining factors express differences between intercept and means for: males in control group (sex factor), females in experimentally manipulated group (experiment factor) and males in experimentally manipulated group (the interaction term).

| Trait | Fixed term | Estimate | 95% CI |
| --- | --- | --- | --- |
| Brightness | Intercept | 66.94 | (65.60; 68.27) |
|  | Experiment | -1.69 | (-3.54; 0.19) |
|  | Sex | -1.16 | (-1.83; -0.27) |
|  | Experiment × Sex | 1.18 | (0.14; 2.35) |
| Saturation | Intercept | 62.59 | (60.92; 63.57) |
|  | Experiment | -2.14 | (-3.72; -0.15) |
|  | Sex | -0.78 | (-1.52; 0.04) |
|  | Experiment × Sex | 0.73 | (-0.36, 1.72) |
| Hue | Intercept | 42.68 | (42.06; 43.44) |
|  | Experiment | -1.40 | (-2.26; -0.44) |
|  | Sex | -0.48 | (-0.83; -0.04) |
|  | Experiment × Sex | 0.89 | (0.22;1.34) |

Table S3. DIC-based model selection for univariate analyses. For each response variable (columns) a set of all possible models is presented together with respective DIC values, rounded to integers. Full models contained random effects of nest of rearing, nest of origin and dyad. Models indicated as “-X” lack the X effect. Best-fitting models are indicated in bold.

| Model structure | Brightness | Saturation | Hue |
| --- | --- | --- | --- |
| Full | **3130** | **3116** | **2321** |
| -Origin | 3146 | 3151 | 2329 |
| -Origin  -Rearing | 3148 | 3178 | 2341 |
| -Origin  -Rearing  -Dyad | 3148 | 3210 | 2361 |
| -Dyad | 3140 | 3167 | 2353 |
